# A Low-Cost, Social Media–Supported Intervention for Caregivers to Enhance Toddlers’ Language Learning: Mixed Methods Feasibility and Acceptability Study

**DOI:** 10.2196/66175

**Published:** 2025-06-23

**Authors:** Mollie Romano, Diana Abarca, Frances Baehman

**Affiliations:** 1 School of Communication Science and Disorders Communication and Early Childhood Research and Practice Center Florida State University Tallahassee, FL United States

**Keywords:** social media, language development, toddler, infant, infancy, pediatric, language learning, feasibility, acceptability, caregiver, TikTok, mixed methods, short video, qualitative analysis, child support, health information

## Abstract

**Background:**

Widely accessible, cost-effective early language development interventions for caregivers of young children are needed to promote optimal outcomes in children in the United States. Social media short-form videos, such as those on TikTok, may be a natural fit for delivering this type of intervention.

**Objective:**

This study aims to examine the feasibility and acceptability of a low-intensity, short-term social media intervention for caregivers of young toddlers.

**Methods:**

In total, 25 caregivers of children aged between 12 and 18 months participated in this study. We shared 32 short-form videos via TikTok over an 8-week period to help increase caregivers’ knowledge about early childhood communication. We examined metrics to characterize participant engagement, explored measures of changes in caregivers’ knowledge, and conducted a qualitative analysis of caregiver interviews after the intervention.

**Results:**

Results indicated that most caregivers were able to consistently view the videos, with approximately 75% (16/21) viewership per video (mean 15.75 likes out of 21 possible likes), and caregivers reported positive effects of the intervention on their knowledge of how to support their child’s communication. The results of the exploratory measure of change in caregiver knowledge were positive but not statistically significant (*t*_21_=–1.357; *P*=.09). Caregivers offered suggestions for content and enhancements to videos for future investigations.

**Conclusions:**

Low-cost, short-term social media interventions could be an effective means to equip caregivers with the information they need to advance their children’s language abilities, particularly for families from lower-income backgrounds whose access to health information about their young children may be limited.

## Introduction

### Background

Infants undergo multiple developmental transitions that change the way they engage with the world around twelve months of age. Most infants take their first steps to become new walkers, become more consistent and advanced gesture users, and make the transition to symbolic language as they say their first words [1]. In the months that follow, variability in children’s expressive vocabulary appears as some children learn words quickly, while others learn words more slowly [2]. Because children’s language outcomes are directly linked to their academic, social-emotional success, and mental health, promoting optimal language learning is a critical public health issue [3]. Efforts to bolster early language learning by supporting caregiver-child interactions, especially in lower-income communities, are key to promoting school readiness, social and emotional learning, and optimal language outcomes in all children [4].

However, more research is needed on effective interventions for caregivers and children at the population level to promote optimal child language outcomes while providing targeted support for children in greater need of intervention [3]. Investments in population-wide, universal interventions may bolster child outcomes by increasing caregiver knowledge and confidence in supporting their infant’s early learning. Unlike more intensive coaching approaches that are often geared toward lower-income families, widely accessible interventions may also avoid stigmatizing families who are often the recipients of preventive interventions [5]. In this feasibility and acceptability study, we describe the development and the use of a cost-effective, media-supported approach to sharing information and strategies with caregivers to support language learning in toddlers aged between 12 and 18 months, a critical developmental inflection point. The BabyTok Project is designed as a light touch social media–supported intervention to increase caregivers’ knowledge about early language learning and how they may promote it with the use of specific strategies.

### Factors That Support Early Language Development

Caregiver-child interactions in the first few years of life are influenced by a family’s culture [6] and what caregivers know and believe about children’s language and cognitive development [7]. Caregivers who believe that children’s language learning begins early and is dependent on the input from adults around them are more likely to engage their children in back-and-forth exchanges [7]. For their part, children also influence caregiver-child interactions through their own initiations and contributions during exchanges through vocalizations, gestures, gaze, and early word use [8].

While it is well established that sufficient language input is related to infants’ early language learning [9], other characteristics of caregiver-child interactions contribute to meaningful moments of early word learning. For instance, the degree to which caregivers and children engage as conversational partners during back-and-forth turn-taking exchanges relates to children’s later language outcomes as well as their neuroanatomy and physiology [10,11]. Caregiver input that is timely, contingent, and functional within the immediate contexts of everyday life also helps to facilitate early language learning [12]. During infancy and the early toddler years, linguistic features such as caregivers’ use of parentese (marked by more exaggerated intonational patterns and repetition of words and phrases), gestural input [13], and touch cues also support early language learning in the first 2 years of life [14]. While some caregivers may know that these strategies support their child’s ability to talk, others may not, particularly if they are first-time caregivers who do not have extensive experience interacting with infants and toddlers or if they do not have access to information about how children learn [7,15].

### Interventions to Increase Caregivers’ Knowledge About Early Language Learning

Several interventions that center on infants, toddlers, and caregivers demonstrate promise in supporting child language learning, particularly in communities experiencing poverty. For example, the “3Ts” home visiting curriculum [7] and another model named Duet [16] use home visiting models to enhance caregivers’ knowledge and responsive interactions with toddlers. However, limitations of such approaches relate to scalability and cost. Training and employing home visitors for more time-intensive models limit the ability of programs to offer support for every child and family who qualifies. This does not mean that more intensive programs are not worth the investments they require; rather, additional approaches are needed at the population level to support widespread access to developmental information that may influence caregiver-child interactions.

Several mobile health interventions aimed at caregivers of very young children have shown evidence of effectiveness in increasing caregiver knowledge [17,18]. For example, an intervention called Sharing Stories used WhatsApp as a platform to promote responsive caregiving and parent well-being within a 6-week intervention in Tanzania. Caregivers in the intervention condition, which consisted of 6 content webinars on early learning, reported higher developmental outcomes for their children than the control group [19]. Fully asynchronous app-based interventions such as Háblame Bebé are also being tested in the United States to examine whether low-intensity interventions have a direct impact on caregiver-child interactions by sharing information about “language nutrition” in the early years of life [20]. While data from a randomized controlled trial are mixed related to the ability of Háblame Bebé alone to impact caregiver-child interactions, caregivers report positive outcomes related to increased knowledge about children’s language learning and cultural pride regarding their use of Spanish with their child [21]. Compared to other areas, such as maternal and child health, developmental interventions that focus on early language development using social media are rare [17].

Although research evaluations to support early language development on social media are scarce, they could be a natural fit for mobile health interventions because many caregivers in the United States use social media to seek information and connections with others as they transition to parenthood [22,23]. Approximately 89% of new caregivers report using social media apps to search for information and social support about caregiving-related topics, such as infant sleep, feeding, growth, and development [24]. Low-income communities in the United States report the use of social media and the internet to access parenting information and social support at high rates, making it a logical platform to share information that could be accessible for all caregivers [23,25]. Mothers with low incomes in the United States have described using their mobile phones as a primary source of access to the internet, and they report using search features to quickly gain access to information while multitasking with other responsibilities [25]. Creators on social media already generate publicly available content specific to supporting language learning in young children and have large followings of caregivers. However, few investigations explore whether caregivers can consistently access and retain information and use strategies from social media to support language-rich interactions with their children.

### BabyTok Project

We first developed and tested the BabyTok Project as a means to connect with teachers of infants and toddlers during the COVID-19 pandemic because it made use of mobile technology while social distancing [26]. In our initial study, we conducted an 8-week intervention with teachers serving infants and toddlers in low-income communities by giving an overview of early communication development in the first year of life, responsive strategies to support child communication in the classroom, and affirmations for teachers about their critical role as early educators during the pandemic. The BabyTok Project reinforced the infant toddler teachers’ positive interactions with children as described by the teachers, encouraged them to implement new communication strategies with children in their classroom in some cases, and increased or reinforced feelings of pride in their role in children’s learning. Results also indicated positive, though not statistically significant, gains in knowledge scores related to early language learning on a standardized measure. Most participants endorsed the use of social media as a platform for the BabyTok Project because of its convenience, engaging style, and short-form nature [26].

### Purpose of This Study

The purpose of this investigation is to examine the feasibility and acceptability of the BabyTok Project as a means to support home-based caregivers’ knowledge about early language development and intervention. Feasibility is characterized by caregiver retention and caregiver engagement with the project videos and procedures, and acceptability will be described through qualitative analysis of caregiver interviews after the intervention. We also use an exploratory measure to determine the potential of the tools to capture change from a light-touch intervention such as the BabyTok Project for future effectiveness studies.

## Methods

### Participants

Study recruitment occurred in the summer of 2022 through an informational video that was posted on TikTok (ByteDance) and the study flyer posted on relevant Facebook (Meta Platforms, Inc) groups. To be eligible, caregivers had to (1) have at least 1 child aged between 12 and 18 months and (2) receive at least 1 public benefit (Women Infants and Children, Medicaid, Temporary Assistance for Needy Families, and housing assistance), which served as a proxy for income. Interested individuals were directed to a survey link that included 2 screening questions to verify their eligibility status (ie, “How old is your child?” “Does your family receive any of the following assistance: commodities, food stamps, housing assistance, Medicaid, supplemental security income, and/or WIC?”). While the intervention was intended to be universal, we focused recruitment on caregivers who used social services to ensure that the intervention was accessible and acceptable to caregivers with fewer financial resources.

After duplicates and incomplete responses from the initial interest survey were removed, 42 eligible caregivers across the United States expressed interest and shared their information to join the study within the 7-day recruitment period. Of them, 27 (64%) caregivers completed pretest measures and began participation in the study, while the remaining 15 (36%) did not respond to pretest measures. We held an initial Zoom (Zoom Communications, Inc) meeting with each of the 27 participants, which ensured the participants were real and not bots. Moreover, 2 (7%) participants did not complete any posttest data and were considered lost to attrition, so demographic data for 25 participants were reported. The study participants included 24 (96%) mothers and 1 (4%) grandmother. In total, 11 (44%) participants were White, 8 (32%) were Black, 2 (8%) were multiracial (Black and White: n=1, 4%; Black, White, and Hispanic: n=1, 4%), 1 (4%) was Asian, and 1 (4%) was Hispanic. In addition, 1 (4%) participant did not self-report her race or ethnicity. All (n=25, 100%) participants spoke English, and 4 (16%) spoke an additional language (ie, Spanish or Vietnamese), and they were aged between 23 and 49 years. In total, 15 (60%) participants reported having a male child, 10 (40%) reported having a female child, and none (n=0, 0%) were identified with a developmental condition (Table 1 presents more demographic information about the participants).

**Table 1:** Caregivers’ demographic information (N=25).

Characteristic		Participants, n (%)
**Relation to the child**		
	Mother	24 (96)
	Grandmother	1 (4)
**Age (y)**		
	20-29	8 (32)
	30-39	15 (60)
	40-49	2 (8)
**Race or ethnicity**		
	Asian	1 (4)
	Black	8 (32)
	Black and White	1 (4)
	Black, White, and Hispanic or Latino	1 (4)
	Hispanic and Latino White	1 (4)
	Hispanic and Latino non-White	1 (4)
	White	11 (44)
	Did not report	1 (4)
**Languages spoken**		
	English	25 (100)
	Spanish	3 (12)
	Vietnamese	1 (4)
**Educational level**		
	High school diploma	4 (16)
	GED^a^ or an alternative credential	1 (4)
	Trade and vocational school	2 (8)
	Some college	5 (20)
	Associate’s degree (associate of arts and associate of sciences)	3 (12)
	Bachelor’s degree (BA and BS)	9 (36)
	Doctorate (PhD)	9 (36)
**Estimated household income (US $)**		
	<10,000	3 (12)
	10,000-19,999	1 (4)
	20,000-29,999	3 (12)
	30,000-39,999	7 (28)
	40,000-49,999	2 (8)
	50,000-59,999	3 (12)
	60,000-69,999	1 (4)
	70,000-79,999	2 (8)
	90,000-99,999	1 (4)
	≥100,000	1 (4)
	Prefer not to answer	1 (4)
**Child’s sex**		
	Male	15 (60)
	Female	10 (40)
**Child’s daytime setting**		
	Childcare program	5 (20)
	Parent’s home	19 (76)
	Another family member’s home	1 (4)

^a^GED: General Educational Development.

### BabyTok Project Videos

The BabyTok Project videos were developed by the research team using the video production capabilities within the TikTok platform. Most videos featured the first author as a narrator in a conversational style that is common on the platform. All videos were recorded by the first author on her handheld iPhone (Apple Inc). The first video was an introduction by the first author explaining her credentials as a speech therapist and researcher and providing more details about the project. She served as the “narrator” for the videos and is a White female researcher who is also a parent. As 56% (14/25) of our sample was not White, we asked for feedback on her narration and relatability to gain participant input to guide future investigations. While we did not use caregiver feedback to guide the development of this set of videos, we built in feedback opportunities in the postintervention interviews to gather caregivers’ ideas and preferences for content. A total of 33 videos (1 introduction video and 32 content videos) were shared with participants across 4 overarching topics: *using words*, *shared book reading*, *learning in everyday routines*, and *affirmations.* Videos were developed using the best available research on strategies to support early caregiver-child interactions and early development as described in the study by Romano et al [26].

At the midpoint, the first author also offered participants an opportunity to choose between topics to give them a role in content development, and most participants chose to gain more information about routines (Table 2 presents more detailed descriptions of the videos).

**Table 2:** BabyTok content video descriptions (n=32).

Topic	Videos, n (%)	Purpose
Using words	9 (28)	Children learn so much in their first few years of life, and their caregivers have a lasting impact on their development. These videos introduced expectations of milestones at various ages and strategies for supporting children’s language development, such as expanding on their words and providing choices.
Shared book reading	6 (19)	Shared book reading with toddlers is shown to support their language learning and academic success. These videos shared information about the benefits of reading to toddlers, strategies to use during book reading, and choosing appropriate books for toddlers.
Learning in everyday routines	12 (38)	Learning opportunities are plentiful throughout daily routines. These videos shared foundational information about routines and examples of strategies to use during common daily activities, such as getting dressed or mealtime, to facilitate language development.
Affirmations	4 (12)	These videos were shared throughout the intervention as messages of encouragement for caregivers about their role as parents.
Participant Input	1 (3)	In this video the narrator asked participants for input about which topics they would like to learn more about in future videos.

### Study Design

We used a convergent parallel design with an intervention mixed methods framework with a primary focus on qualitative data [27] to investigate the feasibility, usability, and acceptance of the BabyTok intervention for caregivers of young children. We collected qualitative data to support the continued development of the BabyTok intervention and quantitative data to investigate engagement and use of the intervention and its short-term effects and explore the utility of the measures used to describe intervention effects. The data were merged after individual analysis to present results on the holistic effects of the BabyTok intervention.

### Measures

#### TikTok Engagement Metrics

TikTok engagement metrics were gathered to examine the feasibility of the intervention to shed light on how many videos participants viewed and how often they liked and commented on the videos. Public TikTok account owners have access to TikTok Analytics, which tracks metrics, including the number of likes, the number of shares, the number of views, and the average time viewers spent watching videos. However, to maintain a level of confidentiality for participants, we used a private TikTok account, and this precluded us from having access to all TikTok Analytics data. However, we were able to manually see total views for each video, the number of likes, and comments by each participant. To collect data regarding the participants’ use of the BabyTok video series, participants were asked to “like” the videos posted to indicate that they viewed them. To collect additional data about participants’ engagement with videos, caregivers were invited to comment on the videos. Individual responses were tracked for each video by clicking each post and reviewing who liked or commented. After the study was completed, the TikTok metrics were analyzed by recording the total sums of likes and comments per video and the total sum of likes and comments per participant across the 8 weeks of the intervention and calculating the mean, SD, and range for each. The feedback was analyzed by reviewing individual comments made for each video.

#### Caregiver Interviews

Postintervention interviews were used to evaluate the feasibility and acceptability of the intervention from the vantage point of the caregivers. Participants were interviewed in English by a trained graduate research assistant or second author through web-based meetings on Zoom using a semistructured interview protocol. The semistructured interview protocol was adapted from a previous study that investigated a similar intervention with childcare providers [26]. The protocol contained 7 questions about what participants recalled from the BabyTok videos, their feelings about their role as caregivers because of the BabyTok intervention, and the impact of the BabyTok intervention on their interactions with their child. Questions also focused on the suggestions and feedback participants had about the BabyTok videos and if they would like to see content about different topics (Multimedia Appendix 1). Each interview lasted approximately 10 to 15 minutes, was screen recorded, and was transcribed using the Otter.ai platform. Two graduate research assistants reviewed the transcriptions and corrected any errors.

### Exploratory Measure (Survey of Parent/Provider Expectations and Knowledge-Research)

For this study’s purposes, the Survey of Parent/Provider Expectations and Knowledge-Research (SPEAK-R) was used as an exploratory tool to quantify changes in parents’ perceptions of their understanding of the early childhood cognitive and language learning. The SPEAK-R is a 22-item, self-administered questionnaire designed to assess knowledge of and beliefs about early childhood cognitive and language development [28]. The questionnaire consists of 4 multiple-choice items, 14 Likert scale items scored as *definitely true (0)* to *definitely not true (4)*, and 4 Likert scale items reverse scored as *definitely not true (0)* to *definitely true (4)*. The questions focused on six topics: (1) bilingualism, (2) early exposure, (3) media use for child learning, (4) nature versus nurture, (5) sensitivity and responsiveness, and (6) talking and reading [28]. The SPEAK-R survey was developed and tested on multiple population samples that included caregivers with low incomes [15]. The second version (SPEAK-2) showed significant, positive correlations between scores and level of education, receptive language skills, and the quality of language in the home environment. The mean SPEAK-R score reported by its developers was 47.27 in a sample with low-income caregivers [15]. The SPEAK-R indices were proven reliable during field testing with Cronbach α scores of 0.92 [28]. The SPEAK-R was conducted at entry and after the 8-week intervention. The measure was gathered and scored using REDCap (Research Electronic Data Capture; Vanderbilt University), and the data were imported into SPSS (IBM Corp) for analysis.

### Procedures

After the consent process was completed, the research team sent out a step-by-step handout via email and SMS text message with information for completing the pretest measures and accessing the BabyTok study channel. Participants completed a demographic survey and the SPEAK-R online. They also scheduled an observation with a member of the research team, hosted via a teleconferencing platform, to verify that they were not bots and to use it to pilot other potential observational measures. Caregivers were asked to interact with their child as they normally would during a typical routine or activity they would be engaging in during that time, such as playing with toys, book sharing, or caregiving activities, such as snacks and diaper changes.

After all participants completed the pretest measures, the research team began sharing videos with all participants on the social media platform 3 to 5 times per week*.* The videos were posted on the channel, and they were sent to the participants via direct messages. Participants were encouraged to watch each video, like, and comment to verify that they viewed the videos. At the end of this 8-week period, participants completed the posttest measures, which included the SPEAK-R and another recorded caregiver-child observation as well as an independent web-based interview regarding participants’ perceptions of the BabyTok intervention.

### Data Analysis

#### TikTok Engagement Metrics

We used a descriptive quantitative analysis to show means and ranges of the TikTok engagement metrics. We obtained simple counts of likes and comments per video and the number of likes and comments per participant.

#### Caregiver Interviews

The transcripts were analyzed based on the template analysis method as described in the study by Brooks et al [29]. The qualitative coding team included a doctoral student, a graduate-level research assistant, and an undergraduate research assistant. To become familiar with the data, each team member read and reread the transcripts and took general notes on thoughts about overall themes in the data. Next, the team met, discussed the data, took more notes, and began developing preliminary codes based on the interview questions, responses to the questions, and themes from a previous BabyTok Project [26]. The 3 main codes developed at this meeting were *takeaways from BabyTok*, *effects of BabyTok*, and *attitudes or perspectives or feelings about BabyTok,* and each code was assigned a different color. Next, the team split the transcripts among individuals and separately coded quotes within the transcripts by highlighting them in the color assigned to the corresponding code. The team met several more times to ensure the codes effectively described the data the data. Codes that were no longer applicable were removed, codes were reorganized and redefined, and a more accurate template was developed. Specifically, we recognized that the code *takeaways from BabyTok* overlapped with the code *effects of BabyTok.* Because of this overlap, the codes were reclassified, with the code *takeaways from BabyTok* becoming a subcode of the code *effects of BabyTok.* In addition, the code *attitudes or perspectives or feelings about BabyTok* was split into the subcodes *supportive feedback* and *recommendations*. The team also identified several pieces of data that did not fit into the codebook framework but were important because they described the usefulness of the BabyTok content for different caregivers and in different contexts. Thus, the new subcode of *supportive feedback: content is useful for all caregivers* was established. Next, the team looked for disconfirming evidence or null effects. Because very few examples were discovered, instead of a separate code, we decided to include *negative or null effects* as a subcode of *takeaways from BabyTok*. Finally, the team cross-checked each other’s coding by pulling a sample of 50 significant quotes from the transcripts and independently coding each quote to ensure consistency of coding. The final coding template included codes, subcodes, definitions of subcodes, and an example of a quote for each subcode (Table 3 presents the codebook).

To ensure the trustworthiness of the data, the data analysis team took several steps, such as those described by Nowell et al [30]. Our approach included data familiarization, debriefing with the first author, checking for coding reliability to support the credibility of our findings, providing thick descriptions coupled with several examples of direct quotes to increase transferability, and keeping an audit trail of our decision-making process to achieve dependability. By taking these steps, the confirmability of the data was established [30,31].

**Table 3:** Codebook for posttest caregiver interviews.

Theme			Definition		Sample quote
**Effects of BabyTok**					
	Changes in how they interacted with their child	Differences in how the caregiver approached communicating, engaging, playing, and building a relationship with their child or children after participating in the BabyTok intervention		“I feel like I’m a lot more mindful of what I’m doing when I’m interacting with her...I try to repeat phrases more often and kind of pause and let her try to do it as well.”	
	Changes in how they viewed their role as parents	Differences in how caregivers viewed and felt about their impact on their children (eg, how they develop, communicate, and learn) and/or differences in how they felt about their own parenting style or skills		“I was very critical about her not speaking yet and...really worried about it. But then she just...made me feel better...it’s okay if they’re not as far with their language as they’re supposed to be...it helps me,...feel more grounded and not,...stressed out about it as much.”	
	Changes they noticed in their child	Differences in the child’s behavior and communication after trying BabyTok techniques, strategies, and tools.		“We’re introducing ‘POW’...the point, wait, and the wait for them to repeat type thing,...I noticed that my daughter was catching on to things a lot quicker when I was using like some of those resources.”	
	Takeaways from BabyTok^a^	Information that caregivers described as remembering, learning, trying, or as being useful after participating in the BabyTok intervention		“...what I remember most is just labeling everything for them to help them build their vocabulary. So just labeling and giving them wait time to just kind of let it sink in and process.”	
**Supportive feedback**					
	Use of social media platforms	Comments about the convenience and accessibility of hosting the videos on TikTok (eg, did not take a lot of time, could do from anywhere, and familiarity with TikTok)		“...I enjoyed all the content, it was super accessible to have it on TikTok, because it was there, and I could watch it once it was posted when it was convenient to me.”	
	Engagement with other caregivers	Comments about interacting with and relating to other caregivers through the BabyTok platform to feel affirmed and build camaraderie		“...just to hear other people’s opinions,...I’m dealing with the same thing as a mom’ and it was just very enlightening. Sometimes you think like you’re the only one in this situation, but come to find out you’re not...”	
	Content delivery expert	Comments about the narrator in the videos and as the facilitator		“She is very enjoyable to watch. She’s really cute. And she’s really engaging....I enjoyed watching her videos...if you’re wondering whether she’s an appropriate candidate to continue doing videos, she’s great. I loved her.”	
	Trustworthiness	Comments about the credibility of the information shared and/or the participants’ confidence in the source		“...I get a lot of parenting videos...but I don’t know, for a research project,...I definitely felt like I was getting the right information versus you never know exactly what you’re getting...”	
	Content is useful for all caregivers	Comments that the content was useful for new and experienced parents as well as other caregivers like grandparents.		“I think it’s a great resource for all parents,...new and parents that are already experienced in parenthood just to open your mind and broaden your horizon and other ways to help implement teaching resources for your child.”	
**Recommendations**					
	Content suggestions	Suggestions regarding the topics covered throughout the BabyTok project (eg, behavior management, potty training, routines, sign language, and bedtime)		“I guess one thing that I wish was covered a little bit more was routines...Routines and...how we can build in little learning...incorporate learning opportunities into our daily routines...”	
	Delivery suggestions	Suggestions regarding how the BabyTok project is shared, including what social media platforms are used, the format of the video, and how the participants were notified		“If maybe, throughout the project, there was like a email that went out at the end of the week, like, ‘Hey, make sure you watch these five videos.’ So, I knew if I missed one, to go back and find that rather than having to scroll through the whole account,...”	
	Suggestions for the engagement of users	Suggestions regarding how to increase interactions with BabyTok users, including how to facilitate discussion among users and how users could share videos of their children		“If it was more interactive with those that were participating...maybe if there was some type of participation through the process, outside of maybe commenting.”	

^a^Null and negative effects are included here and not as a separate subcode because they were uncommon but still relevant.

### Ethical Considerations

All participants provided implied consent for the institutional review board–approved study (Florida State University 10003010) through an online interest form with a waiver of documentation of consent. Participants received up to US $100 in gift cards for compensation. Compensation was distributed via mail after pretest data collection (US $50) and the social media intervention and posttest data collection (US $50). All participants were issued an alphanumeric code for all data files. One single excel file linked personal information to the code. The linking file and deidentified files are kept on an encrypted Microsoft Teams folder hosted by the university.

## Results

### TikTok Engagement Metrics

In total, 32 videos were shared across the 8-week intervention period. The data were gathered on 21 participants. We excluded 4% (1/25) of the participants who were lost to attrition (ie, did not complete any follow-up data) and 12% (3/25) of the participants who unfollowed the BabyTok page after the project ended. We were unable to gather their data from TikTok after they exited the group. Participants were asked to like a video as confirmation that they viewed it; 24% (5/21) of the participants liked all 32 videos, and most (16/21, 76%) of the participants liked more than half of the videos (mean 23.95, SD 8.99; range 3-32).

To understand which videos garnered the most engagement and discussion from caregivers, we examined the number of likes per video and the number of comments per video. The videos with the most likes (n=18) were videos about milestones for children aged 1 year and those aged 18 months and a video about modeling and waiting when labeling objects. In total, 2 (6%) of the 32 videos received the least number of likes (n=13); one was a video that asked participants’ opinions about which video topics they would like to see posted during the week, and the other was about activities to engage in during a morning routine. Overall, viewership of each video was 76% (16/21); the mean was 15.75 (SD 1.39; range 13-18) likes per video. The number of comments per video ranged from 2 to 14 (mean 6.44, SD 2.68). The video with the greatest number of comments (n=14) was posted on the first day of the intervention and asked caregivers to share the words their babies understand. The video with the second-highest number of comments (n=12) asked caregivers to answer the question, “What’s your baby up to?” The videos with the least number of comments, 2 each, were about coviewing with screens and constructive play.

We also gathered the number of comments per participant to understand more about engagement per participant. The number of comments was unevenly spread among the participants, with 4 (19%) of the 21 participants commenting >20 times and 13 (62%) participants commenting ≤5 times (mean 8.81, SD 8.9; range 1-28). When commenting on videos, participants typically shared their positive feelings toward the strategies suggested. Participants shared their excitement to try the strategies. For instance, about a video called *Three Tips for Book Time,* a participant stated, “I’ll have to try this! Especially the waiting strategy.” About a video describing a strategy of pointing, using 1 word, and then waiting a few seconds for a response, a participant shared, “I’m going to try it tonight when I read to him.” Participants also shared how they were trying the strategies and how their children were responding. When commenting on a video about modeling words instead of pressuring children to say words with several prompts, a participant shared, “I did this with [my] son with ‘banana’ [his favorite food], and he tried to say it!” One participant shared how a motivational video helped her after a challenging time with her child. After viewing the video, *They Come to You,* she commented the following:

I needed to hear this today. After a long night with the babe, sometimes it’s so easy to question my motherhood journey.

Other common responses in the comments included sharing about their babies’ milestones and how they felt affirmed and emoticons of smiles, laughs, and hearts.

### Caregiver Interviews

Participants’ perspectives of the BabyTok intervention were organized into 3 main themes: effects of BabyTok, supportive feedback of BabyTok, and recommendations for BabyTok. Each theme included 3 to 5 subthemes, as displayed in Table 3. In total, 23 (92%) of the 25 participants completed individual interviews following the 8-week BabyTok intervention.

### Effects of BabyTok

#### Changes in How They Interacted With Their Child

In response to how the BabyTok videos impacted their interactions with their children, many (21/23, 91%) participants reported becoming more purposeful, more mindful, more positive, paying closer attention to their child, having more face-to-face interactions, and using strategies to promote communication more often. One participant stated the following:

I feel like I’m a lot more mindful of what I’m doing when I’m interacting with her on trying to do things that she might copy so that she can implement that and understand what I’m doing. I try to repeat phrases more often and kind of pause and let her try to do it as well.

Another participant discussed the change in her phone use when interacting with her child:

I think it made me sit down and do more face to face with her. Get off the phone a little bit...and make sure that we are doing things to help promote her speech and just spend more time with them.

#### Changes in How They Viewed Their Role as Parents

Participants reported changes in how they viewed and felt about their impact on their children’s language development. They also expressed how they felt about their own parenting style or skills. For example, one participant described a change in her confidence and in the importance of her role:

I feel a lot more confident in my role and helping to develop her language development. And her development in general, like, the videos and description of the one-on-one time and the eye-to-eye contact, all of that being kind of brain food or setting them up for success...It made me feel like I had a more important role than I was giving myself previously.

Another participant described how her understanding of the parent’s role in a child’s language development changed:

It kind of...put that into perspective and how I actually need to do that [consistently label] to help him develop, like how critical parents are to developing the language. It doesn’t just come naturally to them from listening.

Other (9/23, 39%) participants expressed their thoughts on their role with statements such as, “What I learned is that basically, we play a huge part in, you know, teaching our kids,” and “...it made me feel more confident in things that I was doing,” and “I feel like I have to remember to do a little bit more teaching than I did, than I have in the past.”

#### Changes Noticed in Their Child

Participants described noticing that their children paid more attention, learned more quickly, and began to copy words. For example, after using a specific strategy from the videos, a participant stated the following:

It seems like still, he pays attention more when I do it. And it’s nice, because it shows that he’s trying to listen, and figure out what I’m saying.

Another caregiver noticed that her child was beginning to say words because of trying a strategy suggested in a BabyTok video. She stated the following:

But she is picking things up like—she did say “cat” while we were doing that after I pointed out cat several times on several pages to her. I wasn’t getting that kind of a reaction or that kind of an interest or response before I did that, because I wasn’t waiting for any kind of a response from her.

Another caregiver described a similar change in her child:

We’re introducing “POW,” I believe it was. Like the point, wait, and the wait for them to repeat type thing, which I thought was really cool, because I noticed that my daughter was catching on to things a lot quicker when I was using like some of those resources.

#### Participant Takeaways

When asked what they remembered most about the BabyTok videos, participants described what they remembered, what they learned, or strategies they tried. Many (19/23, 83%) caregivers listed specific strategies that were shared in the videos. One participant said the following:

What I remember most is just labeling everything for them to help them build their vocabulary. So just labeling and giving them wait time to just kind of let it sink in and process.

Another described learning from the demonstrations of implementing strategies:

I think what I remember the most about the BabyTok videos, were just some of the demonstrations, or I guess...suggestions on what you could do with the baby. So, I’ve blown bubbles, but I thought it was really intimate and kind of cool just to blow the bubbles while the kid was sitting. So, we tried that.

A few (5/23, 22%) caregivers also described retaining content related to the emphasis on daily routines. One caregiver recalled how to talk to her baby during daily activities. She stated the following:

So, I think I definitely learned a lot of routines and when to talk and how to during throughout the day. That was really important for me to try to remember I could fit in throughout the day.

Another remembered learning that routines were specific to each child and their family. She stated the following:

I think probably about setting a routine and how it doesn’t have to be like a normal routine. It just has to be, because most people, they brush their teeth immediately before going to bed. But we have to kind of settle her down after that, because it’s always a fight. And that’s okay. It doesn’t have to be [a] normal routine, it just has to be, this is what you do from day to day.

#### Disconfirming and Null Effects

When asked what they remembered most about BabyTok videos, two participants described some of the strategies in the videos as not being useful. For example, one participant recounted the following about the point, one word, and wait strategy:

The one about the POW, the point, and then, say the word and then wait, I found that I like that a lot. It didn’t really work for me. But I think I’m gonna keep trying.

The other participant stated the following about trying the book reading and screen time strategies:

He doesn’t really like books that much or TV. He just doesn’t, you know, so I guess it’s individualized for child. So, a lot of the book things, like I read him books, but usually he doesn’t pay attention to them. So and then, like the screen time things, again, he doesn’t pay attention to the screen at all.

One caregiver was unable to try the strategies or implement new routines with her child because of her busy work schedule. She said the following:

My schedule has been off. So, most of the days he’s at daycare or he’s been with his dad the past three weeks...So I haven’t really got a chance to spend time with him as much.

### Supportive Feedback

#### Use of Social Media Platform

Participants discussed the convenience and accessibility of being able to watch and learn while scrolling on TikTok. One participant described the following:

You’re on TikTok anyway. So, if you follow them, and they’ll come up on your following page, and, you know, it makes it like, not so stressful, like, you’re not researching it, you know, intently, you’re just scrolling, and I learned a million things. Since I’ve started watching videos, I cooked a meal off it last night. And so like, at the same time, when I’m scrolling, I’m like, “Oh, I just learned something I can do with my baby.”

Others liked the ease of use and the short length of the videos. One participant stated the following:

It’s user friendly. You don’t have to worry about, I don’t know, she would just message us videos, and I just click on them, and it pops right up so fast.

Another stated, “I liked that they were smaller, bite sized pieces, bite sized segments.” However, one caregiver requested longer videos to be interspersed with the shorter videos.

#### Engagement With Other Caregivers

Another element of the social media intervention that participants enjoyed was engaging with other caregivers. For example, a participant stated the following:

So just to hear other people’s opinions, like, “Hey, I’m dealing with the same thing as a mom,” and it was just very enlightening. Sometimes you think like you’re the only one in this situation but come to find out you’re not and plenty of other parents are dealing with the same thing.

#### Content Delivery Expert

Participants also shared their approval of the host and speaker in the videos with comments such as the following:

It’s Mollie, right, that was doing the videos? She is very enjoyable to watch. She’s really cute, and she’s really engaging.

She was very interactive and very responsive, like when we would make comments or have questions. So yes, I really enjoyed that part.

#### Trustworthiness

Participants viewed the content of BabyTok as more trustworthy than information they received from other sources, such as family, friends, and content creators on TikTok. One caregiver commented the following:

I get a lot of parenting videos, but I don’t know, this was specifically for a research project, so I definitely felt like I was getting the right information versus you never know exactly what you’re getting on TikTok.

Another described getting a “better perspective” from the content on BabyTok. She said the following:

Basically I really get my advice from like, parents and people, you know, who already have kids. Um, so it was just different to like, you know, actually, like, get a better perspective and learn more about how to teach babies and toddlers, you know how to speak.

#### Content Is Useful for All Caregivers

Participants described the content as useful for a wide range of caregivers in multiple contexts. One participant commented that BabyTok would help introduce strategies for language development to the other caregivers in her child’s life:

But then watching the videos gave me more ideas and how to help other people in our life, introduce things to her as well. So it’s not just my responsibility, Dad can help and grandma can help and things like that.

Some (9/23, 39%) caregivers described BabyTok as useful for all parents. One caregiver commented the following:

I think it’s a great resource for all parents, like new and parents that are already experienced in parenthood just to open your mind and broaden your horizon and other ways to help implement teaching resources for your child.

Another said the following:

So I think different generational gaps have different styles of parenting. So I think it’s very beneficial for different generations. So, for example, a lot of the things that she was teaching I wasn’t familiar with only because I’m not used to doing it that way. So I think different generations would find this beneficial to view those videos as well.

### Recommendations

#### Content Suggestions

To close out the interviews, the interviewers asked the participants if there was anything they wished was improved or included in the video. Many (16/23, 70%) participants responded with varying ideas for content additions, including more content about routines, baby signing, speech delays, help with bedtime, how to handle challenging toddler behaviors, and pretend play with toddlers. For example, a participant stated the following:

I guess one thing that I wish was covered a little bit more was routines...Routines and how you can build more—how we can build in little learning, like, incorporate learning opportunities into our daily routines is what I’m trying to say.

#### Delivery Suggestions

In addition to recommending changes to content, some (10/23, 43%) participants recommended changes to video formats, how participants were notified of videos to view, and using other social media platforms, such as Instagram. Other suggestions included adding more to the videos to make them grab and hold attention. For instance, one caregiver said, “I think it would have been more interesting if there was more captions, or pictures and stuff on there,” and another said, “Eye-catching, so instead just like someone talking, there were different varieties.” One participant discussed the use of actual babies in videos to see the activities and strategies demonstrated. Finally, there were recommendations for how participants were notified of videos to view. A participant suggested a method of notification via email. She stated the following:

I think there were a few times where like, I missed a notification...If maybe, throughout the project, there was like an email that went out at the end of the week, like, “Hey, make sure you watch these five videos.” So, I knew if I missed one, to go back and find that rather than having to scroll through the whole account to see if there was one I hadn’t reacted to or liked or whatever.

#### Suggestions for the Engagement of Users

Two (9%) participants wanted more interaction with other caregivers. The following quote from a participant demonstrated this:

I think, yeah, if it was more interactive with those that were participating. It is nice to watch the videos, you can do it on your own time, but maybe if there was some type of participation through the process, outside of maybe commenting.

Another participant suggested the use of Facebook groups for added interaction among participants:

Facebook has groups, so y’all could make a BabyTok group. And then when y’all have videos, maybe you could put like a post that's like a discussion post about the video from October 11. And then maybe people could comment.

### Exploratory Measure (SPEAK-R)

We used a pre-post, paired samples 1-tailed *t* test to assess whether there were changes in caregiver knowledge on the SPEAK-R. A total of 22 caregivers completed the SPEAK-R at posttest measurement. Pretest data on the SPEAK-R had a mean of 48.62 (SD 7.70; range 30-63), and posttest data had a mean of 51.52 (SD 12.94; range 30-69). The 1-tailed *t* test was not statistically significant (t_21_=–1.357; *P*=.09). Some SPEAK-R data were missing at posttest measurement (n=4), indicating a need for additional support for participants to complete it after the intervention. One participant had a notably low posttest score that might have indicated that they completed the measure without responding to all the items.

### Integrated Results

We merged results across the qualitative and quantitative data to understand the feasibility and acceptability of the BabyTok intervention. As was expected with a distal measure there was incomplete alignment between the SPEAK-R items and the content of the BabyTok intervention. Some items on the SPEAK-R (ie, supporting dual language learning, when children begin learning Science, Technology Engineering and Mathskills) were not covered within the project videos, so we would not expect to see participant comments or gains in these areas. However, there was some overlap, specifically across 3 content areas: screen time, caregiver responsiveness, and shared book reading. Our integrated analysis focused on SPEAK-R items, TikTok metrics, and qualitative themes that were related to those 3 content areas (Multimedia Appendix 2 provides a joint display of merged results).

Overall, we found evidence of positive changes related to participants’ knowledge about caregiver responsiveness and shared book reading across all 3 sources of data. Changes in pretest to posttest raw scores on the SPEAK-R items related to caregiver responsiveness (ie, items 26, 34, and 36) indicated that participants gained new knowledge within this content area, as evidenced by increases in the raw scores. A total of 3 (9%) of the 32 BabyTok videos included content related to caregiver responsiveness, and 5 (16%) videos included content related to book reading. Each of these videos, except one (ie, “Myths about Reading”), received at least 17 likes, which was more than the average number of likes across all videos. These videos also received various comments from participants, ranging from 3 to 9 comments. We collected various quotes from participants related to responding to their children and reading with their children across 4 subthemes, indicating that participants learned new strategies, saw changes in their child, and viewed their roles as parents differently in relation to book reading and caregiver responsiveness.

However, changes on the SPEAK-R items related to screen time for young children (ie, 14, 56, 57, and 58) were mixed from before the test to after the test. A total of 2 of the 32 BabyTok videos included content related to screen time; 1 (3%) had 16 likes and 2 comments; 1 (3%) received 17 likes and 9 comments. No (0/21, 0%) participants discussed this content in their interview, indicating that this content may not have been as important to their experience in the BabyTok intervention.

## Discussion

### Principal Findings

This study examined the feasibility and acceptability of the BabyTok Project with caregivers of toddlers while exploring potential outcome measures for future investigations. We wanted to understand how and to what degree caregivers engaged with the BabyTok Project, what they retained from the content, and what caregivers liked or did not like about their experiences with the videos and the platform. Overall, participants expressed positive feedback about the BabyTok content and the short-form, video-based nature of the intervention. Although the distal knowledge measure (SPEAK-R) showed raw score gains but not statistical significance, most (22/23, 96%) participants listed specific strategies that they recalled from the videos and gave examples of using the strategies with their child.

### Caregiver Engagement With the BabyTok Content

In general, we found consistent engagement through likes from most caregivers throughout the project (mean 15.75, SD 1.39 likes/video; viewership: 16/21, 75%). This could suggest that the intervention is well aligned with caregivers who use their mobile devices to access content on social media with regularity and that the content might fit in easily with their use patterns [25]. Two caregivers reported watching the videos when they were sent a direct message, two other caregivers watched them in a batch and “caught up” on the videos when prompted. Still more caregivers viewed the videos from their “For You” screen on the app, suggesting that the multiple points of contact may have helped increase the ability of caregivers to access the videos. The overall viewership and engagement data among caregivers were similar to that of the infant toddler teachers in our earlier investigation (16/21, 75% and 73%, respectively). While it is not possible to directly compare to other app-based studies such as Háblame Bebé that can track time in the app [21], we note similar patterns of engagement in that most (16/21, 76%) caregivers participated at intended levels, with a few low responders, indicating the need for individualized support. One caregiver in our investigation had notably low viewership, suggesting the need for additional strategies to increase engagement for some caregivers. Such individualization could include tailoring when the direct messages are sent (to align with their time on social media or their off time from work) and when, how, and how often reminders are sent.

### Caregivers Gave Specific Details From the Content

Caregivers described the convenience of the short, “bite-sized” content units, that they were on the app anyway for other purposes, such as looking for dinner recipes or entertainment, and that they were able to learn useful information to support their child’s learning. Caregivers noted they may not have had time to watch long videos, but the short content units kept them in touch with the ideas presented in the videos more often and made it enjoyable. This feedback was also consistent with that of the infant toddler teachers in the earlier investigation. Although this study did not experimentally evaluate whether caregivers made changes in their interactions, caregivers did indicate that they believed the approach supported them in engaging their child in language-rich interactions and equipped them with strategies to use every day. Among the strategies, caregivers found the content related to the point, one word, and wait strategy to be salient, along with expansions and book-sharing strategies for their toddlers. The specificity of caregivers’ responses around these strategies was encouraging because it reflected ideas shared in the videos that caregivers were not likely to have learned elsewhere.

### Caregivers Described the Trustworthiness of the Content

The first author, who is also the main video narrator, provided her credentials in an introductory video at the beginning of this study. This may be one reason that caregivers described the content viewed on the BabyTok page as “the right information” and that it gave them a “better perspective” about toddler language development than information they received from family, friends, and other TikTok creators. These statements relating to trustworthiness are encouraging and may indicate caregiver acceptability of the content and overall intervention.

### Suggestions for Additional Content

Caregivers gave suggestions for future videos, which can be implemented into future studies, such as including the use of different methods of delivery (ie, Instagram or Facebook groups); use of text on screen, including more eye-catching images; and videos with real babies and caregivers to show examples of the strategies. They also suggested content that extends from communication content directly into everyday concerns, such as sleep and challenging toddler behavior. This is important because caregivers seem to not only want information about how to help their children talk but also ways to navigate everyday challenges that caregivers of children in this age range face, which may then impact their interactions with their child. Tackling these parenting issues may also give caregivers a means to engage with one another as they share what has worked and what has not for their child, which could serve to build their own confidence in sharing their experience with one another.

### Implications for Media-Based Universal Interventions

While we cannot extend these findings to other types of interventions, there are potential implications for video-based interventions with caregivers of young children. Similar to our experience with teachers [26], home-based caregivers did not have a 100% watch rate, but they did view most videos, on average. Because few caregivers will be able to watch every video, it is important to build in redundancy by reinforcing similar messages and delivering the same message more than once. Furthermore, our attempts at reaching caregivers through multiple modes, such as sending videos via direct messaging, SMS text message reminders, and the “For You” page. seemed to help increase the number of videos viewed by each caregiver. While publicly available content may have similar messages as the BabyTok videos, there are no mechanisms yet to support caregivers to consistently engage with the content beyond what appears based on the algorithm or to ensure that enough video content reaches users to create changes in knowledge and caregiver-child interactions.

### Limitations and Future Directions

While the findings in this investigation suggest the promise of social media–based interventions to support caregiver knowledge about early language learning, the study does face several limitations. First, the small sample size of participants may be a limiting factor in evaluating and drawing conclusions from the data. Next, because of the nature of the TikTok platform, we had limited metrics available to us as a private TikTok page. With this lack of metrics, we were not able to gather data on the watch time of each video by the participants. It is possible that participants may have pressed “like” on the video without fully viewing it, and it is also possible that participants did view the video but neglected to press “like,” leading to an underrepresentation of their viewing. We also did not collect data to understand how our participants typically engaged with and used social media to compare with how they engaged with and used the BabyTok content relative to other social media content. We made assumptions that participants engaged with content if they liked and commented on the videos, but they may have engaged in other ways, such as showing the video to a friend or partner.

In future studies, we could ask caregivers to report on their overall social media use patterns to compare their engagement with the content relative to their overall social media use. Although our sample of participants was similar in age and gender to the average US TikTok user, there were some differences in parenting status, education level, income level, and race or ethnicity [32,33]. These differences may have made it more difficult to evaluate engagement data of some participants, as they may not have been typical TikTok users. Our participants were more educated (bachelor’s degree: 9/25, 36%) compared to the average US adult aged >25 years (23.5% with a bachelor’s degree) [34], and fewer of our participants had their toddlers in childcare outside the home (6/25, 24%) than the average US family (55%) [35]. This may indicate that caregivers who are in the home and more educated may be more likely to access and engage in this type of social media intervention.

Although some caregivers reported gains in their child’s communication, we did not directly measure child language skills as an outcome in this feasibility and acceptability study. It is important to note that children should acquire new words during this developmental period, so randomized controlled trials are needed to determine whether there are child-level effects above and beyond the communication gains that would take place through the child’s maturation alone.

The SPEAK-R provided a related outcome representing caregiver knowledge, although there were items on the measure that did not align with the video content (ie, early mathematics and cognitive concepts). Those domains would not have been likely to change because of the BabyTok intervention. SPEAK-R has been used as an outcome in a few studies that offer video-based educational content [36], but it is difficult to compare relative changes between studies because our intervention focuses only on communication rather than communication and cognition. In addition to measuring caregiver knowledge, it would also be useful to measure long-term impacts on children’s vocabulary development by using caregiver-reported tools about their child’s communication in a larger, randomized study. Additional future directions also include creating and testing Spanish-language videos for caregivers in the United States who speak Spanish as a primary language, with a focus on supporting children in both languages.

### Conclusions

The findings of this study add evidence to the feasibility and acceptability of the BabyTok Project as a means to increase caregiver knowledge of how to support children’s language learning. This research is important because, to date, there have been few social media–based interventions targeting children’s early communication. While there is a sizeable body of evidence related to intensive coaching models to help caregivers engage with their children, low-cost, light-touch interventions could be a scalable and accessible means to equip caregivers with information they need to advance their children’s language abilities [21], with downstream effects on academic and social outcomes if future efficacy testing indicates positive effects. This approach to intervention may be particularly important for families from low-income backgrounds whose access to health information about their young children may be limited.

## Appendix

Multimedia Appendix 1 Semistructured interview protocol.

Multimedia Appendix 2 Joint display of merged results.

## Abbreviations

REDCap: Research Electronic Data Capture
SPEAK-R: Survey of Parent/Provider Expectations and Knowledge-Research
