# Supplementary material for: A Low-Cost, Social Media–Supported Intervention for Caregivers to Enhance Toddlers’ Language Learning: Mixed Methods Feasibility and Acceptability Study
Source: JMIR Pediatr Parent. 2025 Jun 23;8:e66175. doi: 10.2196/66175 (PMC12235199; doi:10.2196/66175)
Supplement: Multimedia Appendix 1 [file pediatrics_v8i1e66175_app1.docx]

Post Interview Questions- Babytok 2.0

- What do you remember most about the BabyTok videos?
- Tell us about what new things you learned, if any, from the project.
- Can you think of any ways that the videos changed how you feel about your role as a parent in supporting your child’s language?
- Were there any topics that you wish we had elaborated further on?
- What would you tell others who might be interested in participating in the BabyTok Project?
- Were there parts of the experience you wished were a little different or that could be improved?
- How do you think the videos impacted your interactions with your child?
