# Supplementary material for: A Low-Cost, Social Media–Supported Intervention for Caregivers to Enhance Toddlers’ Language Learning: Mixed Methods Feasibility and Acceptability Study
Source: JMIR Pediatr Parent. 2025 Jun 23;8:e66175. doi: 10.2196/66175 (PMC12235199; doi:10.2196/66175)
Supplement: Multimedia Appendix 2 [file pediatrics_v8i1e66175_app2.docx]

| **Screentime for Young Children** | | | | |  |
| --- | --- | --- | --- | --- | --- |
| SPEAK-R Items | | | | |  |
| 14. Showing infants educational TV gives them a jump-start on learning how to talk **(1.095 - 1.095)** | | 56. Children 0 to 2 years old can learn just as many words from educational TV as they can from their parents. **(1.476 - 1.714)** | | 57. Leaving the TV on in the background is a great way to give 0 to 2 year olds extra chances to learn words. **(1.333 - 1.286)** |  |
| TikTok Data | | | | |  |
| Video: Screen Time Part 1 | 17 likes, 9 comments  Comment Examples:   - *She does FaceTime family that lives far away from us with her big sis.* - *Most of the time, she’s watching whatever big sis is. Like bluey, Peppa pig, Mickey Mouse clubhouse.* - *Definitely video calls and for entertainment we do shows that aren’t overstimulating and contain more realistic and real word material like Ms. Rachel.* | | | |  |
| Video: Co-Viewing with Screens | 16 likes, 2 comments  Comment Examples:   - *Yes we do this with any shows like ABCs (I'll practice signing the letters and asking her to repeat the sounds) or with Dora pointing out the colors* - *We do this with Bluey* | | | |  |
| **Caregiver Responsiveness** | | | | | |
| SPEAK-R Items | | | | | |
| 26. When infants babble, parents should respond as if the infant is saying real words. **(2.7612.905)** | | | 34. Answering only if a toddler uses words instead of just pointing better helps the toddler learn how to talk**. (1.429 – 2.048)** | | 36. Answering only if a toddler uses correct words for things instead of baby words (like "blanket" instead of "ba-ba") better helps the toddler learn how to talk. **(1.667 - 2.143)** |
| TikTok Data | | | | | |
| Video: Expansions! Language Level Up | | 17 likes, 5 comments  Comment Examples:   - *My son signs better than talking he’s 13 months. So when he signs ‘more’, I sign and say ‘more please’. Hopefully he will speak more soon.* - *We do this. My daughter will say ‘Up’ and I'll add ‘Up please’ or say ‘I would like to get up’. We also talk about colors or shapes of things like ‘Red ball’* | | | |
| Video: Try this, not that! Prompting | | 18 likes, 9 comments  Comment Examples:   - *Sooo guilty of the mama thing. He says dada and buhbuh for his dad and brothers, so mama feels left out lol.* - *Ooh I never really thought of this, definitely have to try that!* - *I did this with son with ‘banana’ (his favorite food) and he tried to say it!* | | | |
| Video: What Should my 18-month-old do? | | 18 likes, 3 comments  Comment Examples:   - *My 15-month-old tries to say chicken and fishy, it’s super cute. She’s very food motivated 😂.* - *My son calls my nephew Elijah ‘jaja’ when he’s looking for him.* | | | |
| **Shared Book Reading** | | | | |  |
| SPEAK-R Items | | | | |  |
| 37. Letting a toddler move around while listening to a story teaches the toddler bad listening skills. **(2.333 - 2.095)** | | | 39. Letting a toddler skip words and pages teaches the toddler bad reading habits. **(2.143 - 2.286)** | |  |
| TikTok Data | | | | |  |
| Video: Myths About Reading | | 14 likes, 9 comments  Comment Examples:   - *We reread books alllll the time. Very often. My 3.5yo loves reading the same book and always has.* - *My son would have to be sitting in my lap to learn.* - *We have ALOT of books. My oldest is 5 and loves to read so for story time we switch each day on who can pick out the book so we don’t re-read.* | | |  |
| Video: Trouble Shooting Book Time | | 1. likes, 6 comments   Comment Examples:   - *. Great idea, I have to catch mine being still only for a few minutes.* - *Highchair is a GREAT tip wow!* - *We read books in bed before naptime.* | | |  |
| Video: Books are for Babies Too! | | 17 likes, 4 comments  Comment Examples:   - *I love reading to my daughter and I try to squeeze it in at least a couple times in the day!* - *I’ve heard that reading adds vocabulary and all it takes is 20 mins. I’ll have to try to find the statistic! Mind blowing! :)* | | |  |
| Video: 3 Tips for Book Time | | 17 likes, 7 comments  Comment Examples:   - *I’ll definitely have to try this. I do it some, but could be better about it, especially not reading all the words and just freestyling it.* - *I’ll have to try this! Especially the waiting strategy and the expansion of what’s going on* | | |  |
| Video: The Art of Books that Babies Love | | 17 likes, 7 comments  Comment Examples:   - *Never thought about rhyming like that before. Time to look through our books!* - *My son loves books that have a noise component to it!* | | |  |
